# Insecticidal efficacy of fluralaner (Bravecto^®^) against *Triatoma brasiliensis*, a major vector of *Trypanosoma cruzi* in Brazil

**DOI:** 10.1186/s13071-021-04978-x

**Published:** 2021-09-06

**Authors:** Tamyres Bernadete Dantas Queiroga, Luanderson Cardoso Pereira Gomez, Eduardo Rodrigues de Sena, Wilo Victor dos Santos, Henrique Rafael Pontes Ferreira, Vicente Toscano de Araújo-Neto, Andressa Noronha Barbosa-Silva, Carlos Ramon do Nascimento Brito, Romeika Karla dos Reis Lima, João Ciro Fagundes-Neto, Lúcia Maria da Cunha Galvão, Henrique Rocha de Medeiros, Antônia Cláudia Jácome da Câmara, Manuela Sales Lima Nascimento, Renata Antonaci Gama, Paulo Marcos Matta Guedes

**Affiliations:** 1 Graduate Program in Pharmaceutical Sciences, Federal University of Rio Grande Do Norte, Natal, Rio Grande Do Norte Brazil; 2 Graduate Program in Parasitary Biology, Federal University of Rio Grande Do Norte, Natal, Rio Grande Do Norte Brazil; 3 Department of Clinical and Toxicological Analyses, Federal University of Rio Grande Do Norte, Natal, Rio Grande Do Norte Brazil; 4 Canis and Catus Veterinary Clinic, Natal, Rio Grande Do Norte Brazil; 5 Zoonoses Control Center, Natal, Rio Grande Do Norte Brazil; 6 Agricultural School of Jundiaí, Federal University of Rio Grande Do Norte, Macaíba, Rio Grande Do Norte Brazil; 7 Department of Microbiology and Parasitology, Federal University of Rio Grande Do Norte, Natal, Rio Grande Do Norte Brazil

**Keywords:** Chagas disease, Control, Fluralaner, Bravecto^®^, Systemic insecticide, *Triatoma brasiliensis*, Dog, *Trypanosoma cruzi*

## Abstract

**Background:**

Triatomines are responsible for the vector transmission of the protozoan parasite *Trypanosoma cruzi*, which causes Chagas disease. *Triatoma brasiliensis* is the main vector of the parasite in Brazil, and dogs are an important reservoir of the parasite. The aim of this study was to evaluate the insecticidal effect of fluralaner (Bravecto^®^) on *T. brasiliensis* after a blood meal in treated dogs.

**Methods:**

Healthy mongrel dogs (*n* = 8) were recruited from the Zoonoses Control Center (ZCC) in the city of Natal, Rio Grande do Norte, Brazil, and randomized into two groups, a fluralaner (Bravecto^®^)-treated group (*n* = 4) and a control group (*n* = 4). Colony-reared third-, fourth- and fifth-instar nymphs of *T. brasiliensis* nymphs (*n* = 10) were allowed to feed on dogs from both groups for 30–40 min, once monthly, for up to 12 months. Bug mortality was observed up to 5 days after each blood meal.

**Results:**

Mortality in triatomines which had a blood meal on fluralaner (Bravecto^®^)-treated dogs was 100% for up to 7 months after treatment, with mortality decreasing to 66.4% after 8 months, 57% after 9 months, 35% after 10 months, 10% after 11 months and 0% after 12 months. The mortality of triatomines that fed on non-treated control dogs was always ≤ 2.5%.

**Conclusions:**

Our results suggest that fluralaner (Bravecto^®^) treatment of dogs induces long-term mortality of *T. brasiliensis* after the blood meal. This is a potential approach to be used to control vector transmission of *T. cruzi*, the etiological agent of Chagas disease, especially in endemic areas.

**Graphical Abstract:**

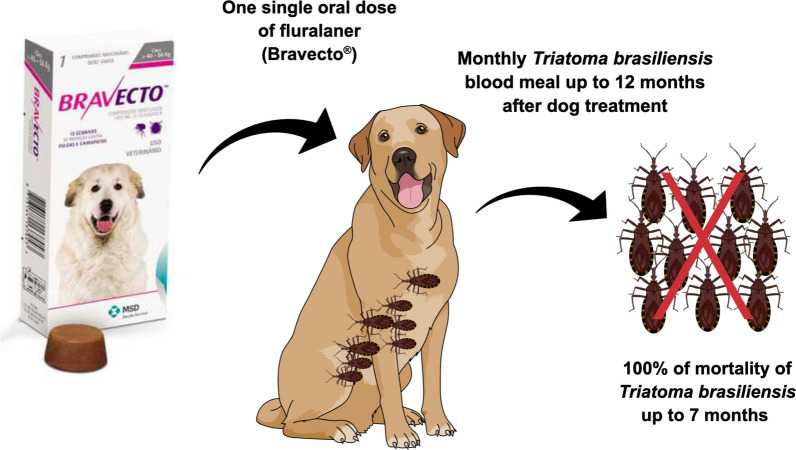

## Background

*Trypanosoma cruzi* is the etiologic agent of Chagas disease. Worldwide, an estimated 6–7 million people are infected with this agent and 75 million people live in areas at risk of contracting *T. cruzi* infection [1]. The main transmission route to humans in Latin America is through blood-sucking bugs (Hemiptera: Reduviidae: Triatominae) by the classical contaminative route consisting of metacyclic forms eliminated by infected triatomines in feces and urine. Several species of triatomine bugs are able to transmit *T. cruzi*, but *Triatoma infestans*, *Triatoma dimidiata*, *Triatoma brasiliensis*, *Rhodnius prolixus* and *Panstrongylus megistus* are considered to be the most important primary vectors of the parasite in Latin American countries [2–4]. Other transmission routes, such as oral and congenital routes, are also important in some endemic countries [1, 5]. It is important to highlight that oral transmission is also vector dependent as infected insects are responsible for food or drink contamination [6].

The use of pyrethroid insecticides in domestic and peridomestic environments is the main or only intervention used to prevent the vectorial transmission of *T. cruzi* by triatomines [4, 5]. Synthetic pyrethroids have currently replaced the use of organochlorine and organophosphate insecticides due to their longer lasting effect and lower toxicity. However, the residual effect of the insecticide remains only for a few weeks in areas surrounding dwellings due to environmental conditions [7, 8]. Thus, epidemiological surveillance and insecticide reapplication are often necessary to avoid recolonization. In addition, the use of insecticides has stimulated the appearance of *T. infestans* populations that are resistant to pyrethroids in Argentina [9], Bolivia [10, 11] and Brazil [12]. Resistance of the triatomine bug *Rodhnius prolixus* to pyrethroids has also been described in Venezuela [12] and Colombia [13]. The solution to combat triatomines in these cases is once again the use of insecticides, namely those with greater adverse effects on man, such as organophosphate insecticides (fenitrothion, malathion) and carbamates [14, 15].

Dogs are a link in the chain between sylvatic and domestic transmission cycles [16–21] and have been described as an important domestic reservoir of *T. cruzi* in several Latin American countries, including Mexico [18, 22], Peru [23], Venezuela [24], Costa-Rica [25], Paraguay [19, 26], Bolivia [27], Argentina [21], Chile [28], Ecuador [29] and Brazil [16, 30]. In addition, *T. infestans* has been reported to have a preference to feed on dog blood than on chicken blood. This preference can be exploited to reduce domestic insect populations by using topical lotions or insecticide-impregnated collars on dogs [31, 32]. *Triatoma brasiliensis* is the most important *T. cruzi* vector in the semi-arid areas of northeastern Brazil, and domestic dogs are also important reservoirs in these areas [16, 33]. Thus, dogs are promising targets for interventions, as preventing their infection also prevents human cases of Chagas disease.

Fluralaner (Bravecto^®^) belongs to the isoxazoline compound class and acts as an antagonist of γ-aminobutyric acid (GABA)-gated chloride ion (Cl^−^) channels, preventing the entrance of Cl^−^ into the postsynaptic neuron, leading to hyperexcitability of the insect central nervous system [15]. Fluralaner (Bravecto^®^) has potent acaricidal activity against ticks and mites, as well as insecticide activity against infestations of phlebotomine sand flies, such as *Phlebotomus papatasi* [34, 35], *Phlebotomus perniciosus* [36] and *Lutzomyia longipalpis* [37], in dogs, cats and poultry [38–42]. Dogs treated with fluralaner (Bravecto^®^) do not experience any side effects [43], and the compound has significant selectivity for insects over mammalian neurons [44]. Recent studies with fluralaner have shown insecticide activity against *T. infestans* after blood meals on fluralaner (Bravecto^®^)-treated dogs [45, 46]. The administration of fluralaner (Bravecto^®^) to dogs housed in experimental kennels generated 100% mortality of *T. infestans* nymphs for up to 51 days post-treatment [24]. In another study, the administration of fluralaner (Bravecto^®^) to dogs residing in rural houses in Argentina generated 100, 81 and 77% *T. infestans* mortality at 60, 90 and 120 days post-treatment, respectively [46]. However, the insecticide action against *T. brasiliensis*, the main vector in Brazil [33], has not yet been determined. In this study, we evaluated the systemic insecticidal activity of fluralaner (Bravecto^®^) against *T. brasiliensis* after blood feeding on fluralaner-treated dogs compared to a control group, with blood-feeding at once-monthly intervals for 1 year.

## Methods

### Dog recruitment and maintenance

Dogs were obtained from the Zoonoses Control Center (ZCC), Natal, Rio Grande do Norte State (Brazil). With permission of the ZCC, eight healthy dogs were recruited and maintained in individual kennels. Water was provided ad libitum, and the amount of food provided corresponded to 5 and 10% of body weight for normal body weight and obese animals, respectively. No insecticide treatment was used on the kennels or dogs during the assays, and the dogs were bathed with neutral shampoo 2 weeks before each assay. All experimental protocols were performed according to the Brazilian National Animal Care Ethical Council and the Ethics Committee on Animal Use of the Federal University of Rio Grande do Norte under protocol number 136.061/2018.

### Study design

All animals weighed 20–50 kg, were aged between 5 and 11 years and were outbred dogs. The dogs were randomized (tossing of coin in the presence of an observer) into two groups: the fluralaner (Bravecto^®^)-treated (*n* = 4) and untreated control (*n* = 4) groups, respectively, with each group comprising two male and two female dogs. The physical condition of the animals was previously evaluated according to Laflamme [47, 48] and the dogs were classified as normal body weight (ribs easily palpable with minimal fat coverage; waist easily seen by looking over the animal; abdomen retracted when viewed from the side) and obese (ribs palpable with difficulty or not palpable; intense fat coverage; fat deposits evident on the lumbar area and base of the tail; waist does not exist; there is no abdominal depression; evident abdominal distention). Each group consisted of two animals of normal body weight and two that were obese. There were no significant differences in the weight or age of dogs between the control and treated groups. All animals in the treated group received a single oral dose of fluralaner (Bravecto^®^); Merck Animal Health, Kenilworth, NJ, USA) on the same day, following the manufacturer’s recommendations. The triatomine feeding assays were performed on both groups at day zero (before treatment, for the treated group) and at once-monthly intervals between months 1 and 12. The study was conducted from December 2019 to December 2020. No side effects among fluralaner (Bravecto^®^)-treated dogs were observed during the study.

**Fig. 1:**
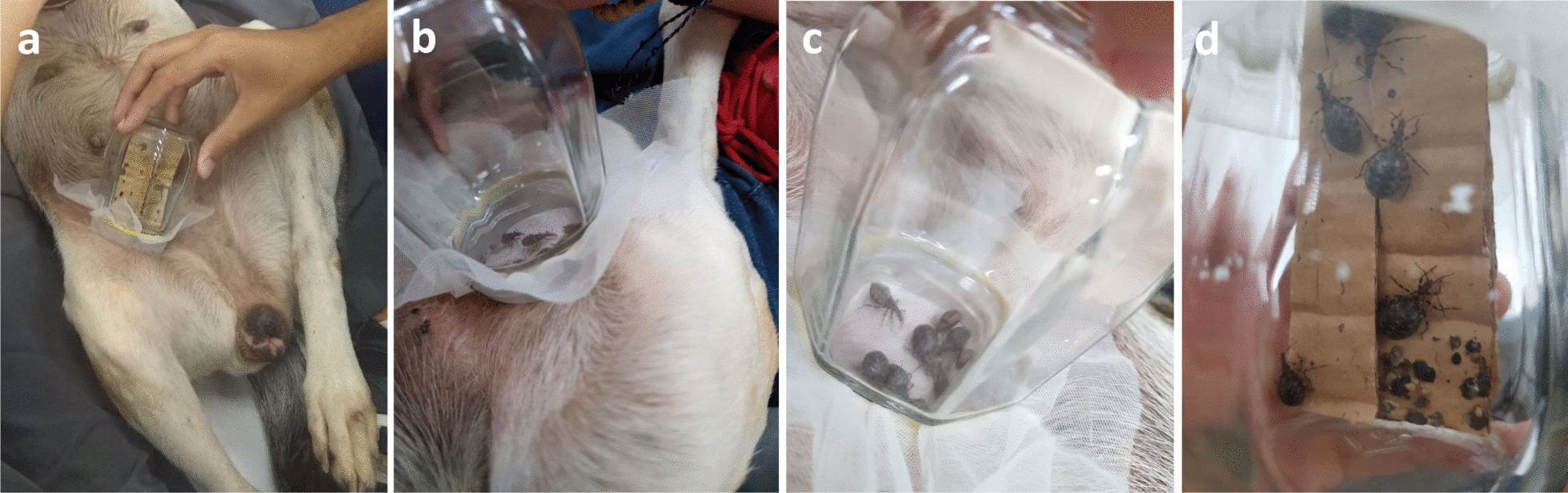
Entomological bioassay.** a** Ten specimens of third-, fourth- and fifth-instar nymphs of *Triatoma brasiliensis* were placed in a glass pot with the top covered with a fine mesh and allowed to blood-feed directly on the abdomen of fluralaner (Bravecto^®^)-treated dogs and on control animals for 30–40 min.** b**,** c** Blood-feeding by insects on a dog from the treated group.** d***Triatoma brasiliensis* after finishing a blood meal. The insects were subsequently maintained in an incubator under standard conditions (28 °C; 50% relative humidity) and monitored at 24, 48, 72, 96 and 120 h after blood meal to determine the mortality rate

*Triatoma brasiliensis* specimens were obtained from the insectary of the Immunoparasitology Laboratory of the Department of Microbiology and Parasitology and the Laboratory of *T. cruzi* Biology and Chagas disease of the Department of Clinical and Toxicological Analyses, both from the Federal University of Rio Grande do Norte-UFRN, Natal, Brazil. The triatomine colony was started in 2012 with insects captured in Rio Grande do Norte State, Brazil. Third-, fourth- and fifth-instar nymphs of *T. brasiliensis*, 10 specimens without blood-feeding (20–30 days before the test), were used in the assays for each animal. Third-, fourth- and fifth-instar nymphs were used due to the low mortality in these stages and the large amount of blood that they can ingest during the blood meal. For blood-feeding, the dogs were gently restrained and positioned upright or lying down for a more comfortable position, following which glass pots containing the insects, with the top covered with a net, were placed on the abdomen of each dog and the insects allowed to to feed for 30–40 min (Fig. 1a–c). The blood-feeding success of the triatomines was checked after the alloted blood-feeding time, and non-feeding insects were removed from the experiment. The engorged triatomines (Fig. 1d) were maintained in an insectary (BOD incubator) under standard conditions (28 °C; 50% relative humidity; dark conditions). Insects were monitored at 24, 48, 72, 96 and 120 h after the blood meal for the analysis. Moribund bugs (insects with impaired mobility) were combined with dead bugs for the mortality evaluation.

### Statistical analysis

The generalized linear mixed model (GLMM) for repeated measures was used to verify the effect of Bravecto^®^ treatment and dog nutritional status on triatomine mortality over time. The model included the fixed effects of Bravecto^®^ treatment, time, dog nutritional status and the interaction between these variables. Differences between groups were considered significant when *P* < 0.05. The analyses were performed using the SPSS version 20.0 software program (SPSS IBM Corp., Armonk, NY, USA) and graphs were created using the PRISM 9.0 software program (GraphPad Software Inc., San Diego, CA, USA).

## Results

### Fluralaner (Bravecto^®^) induces 100% mortality of *T. brasiliensis* up to 7 months after treatment of dog and has insecticidal efficacy for up to 10 months

**Fig. 2:**
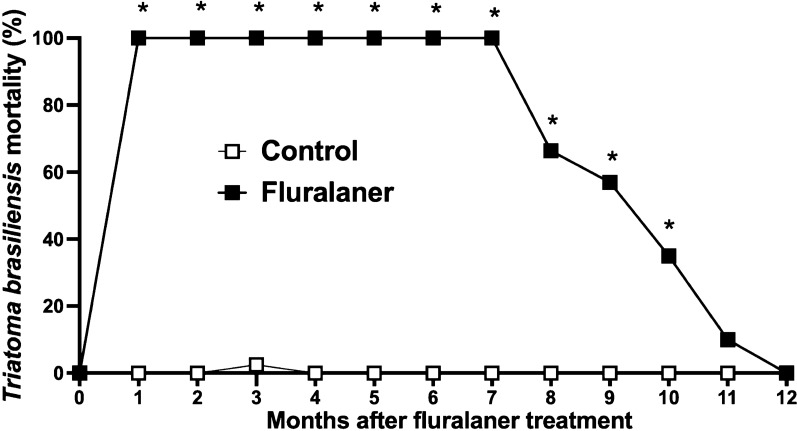
*Triatoma brasiliensis* mortality (%) at 120 h after a blood meal on outbred dogs treated (*n* = 4) and untreated (*n* = 4) with fluralaner (Bravecto^®^). Mortality was assessed before the treatment and once monthly for up to 12 months following treatment with a single oral dose of fluralaner. Fluralaner (Bravecto^®^) induced 100% *T. brasiliensis* mortality for up to 7 months after treatment. Asterisk indicates a significant difference (*P* < 0.05) in mortality between *T. brasiliensis* blood-fed on fluralaner-treated dogs and those blood-fed on control dogs

No side effects were observed in dogs treated with fluralaner (Bravecto^®^) in this study. Using the GLMM for repeated measures to analyze the results over the entire study period, we found that mortality was higher in triatomines that had a blood meal on fluralaner (Bravecto^®^)-treated dogs than in dogs of the control group (*P* < 0.001) (Table 1). In addition, the treatment induced 100% mortality in *T. brasiliensis* up to 7 months (Fig. 2).

**Table 1:** Evaluation of insecticidal efficacy in dogs of fluralaner (Bravecto^®^) against *Triatoma brasiliensis*

Variables	Numerator* df*	Denominator* df*	Z	*P*
Body weight (BW)	2	2.125	180.702	0.247
Bravecto® treatment (BT)	2	52.545	34,070.071	< 0.0001***
Months of treatment (MT)	6	17.600	29,992.552	< 0.0001***
BW × BT	1	3.842	1788.082	0.0782
BW × MT	1	1.745	120.778	0.8313
BT × MT	1	48.111	4392.900	< 0.0001***

Effect of Bravecto^®^ treatment and dog nutritional status on triatomine mortality over time was analyzed using the generalized linear mixed model (GLMM) for repeated measures

***Significan difference between groups at *P* < 0.0001

The insecticidal activity of fluralaner (Bravecto^®^) against *T. brasiliensis* decreased to 66% after 8 months, to 57% after 9 months and to 35% after 10 months; however, the insecticidal action of fluralaner (Bravecto^®^) was always maintained when compared to that of the control group (*P* < 0.001). At 11 and 12 months after fluralaner (Bravecto^®^) treatment, triatomine mortality decreased to 10 and 0%, respectively after the monthly blood meal in the treated dogs, which was not significantly different from mortality in the control group (*P* > 0.05) (Fig. 2). Among the 480 nymphs used to be fed on fluralaner (Bravecto^®^)-treated dogs, only 2.1% (10/480) did not blood-feed; in comparison, 2.5% (12/480) of nymphs in the control group did not feed. These results show that fluralaner (Bravecto^®^) induces long-term mortality of *T. brasiliensis* after a blood meal in treated dogs.

### Time to achieve the full insecticidal efficacy of fluralaner (Bravecto^®^) against *T. brasiliensis* increases according to the treatment period

**Fig. 3:**
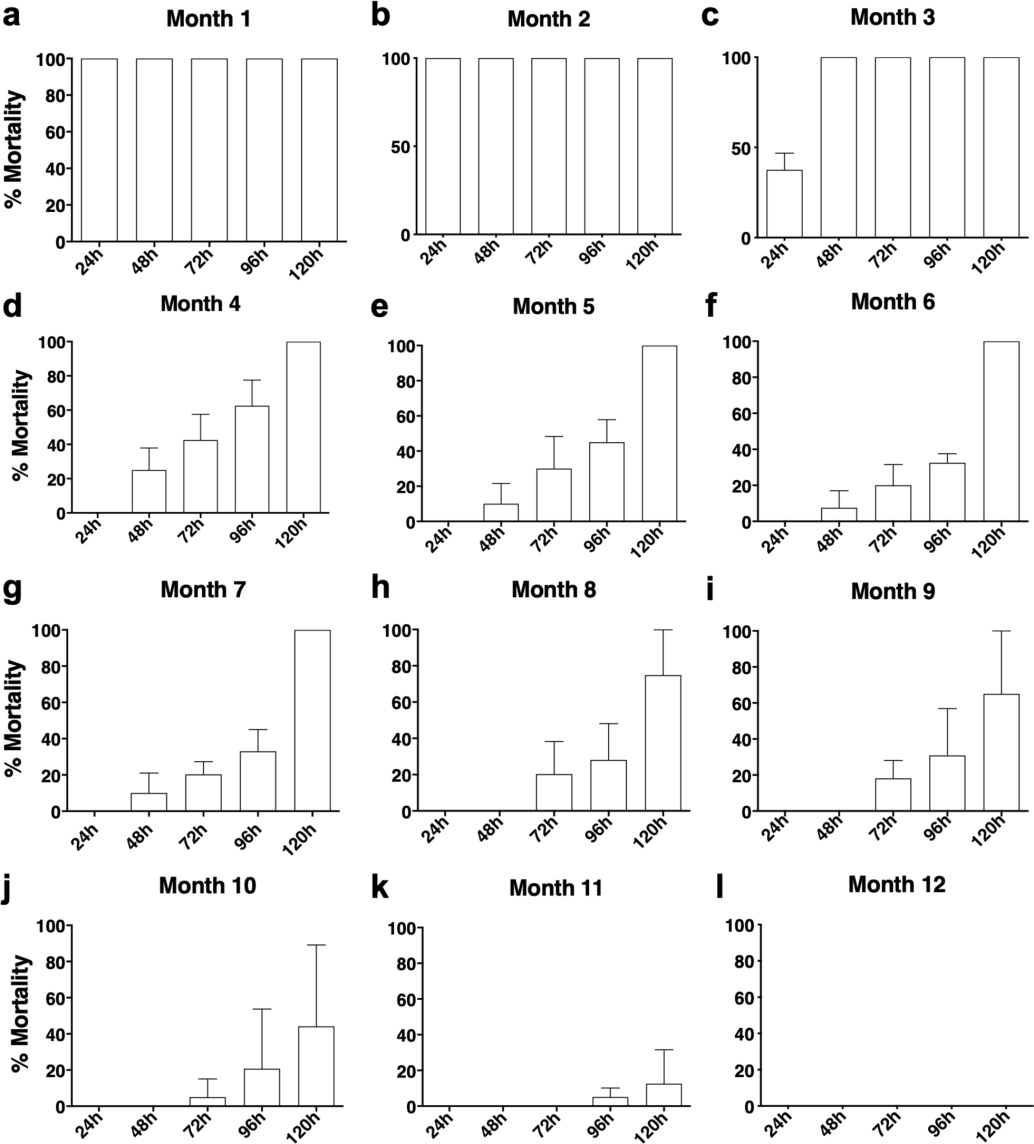
Kinetics of fluralaner (Bravecto^®^) insecticidal action against *T. brasiliensis* blood-feeding on fluralaner-treated dogs. Mortality proportion (%) of third-, fourth- and fifth-instar nymphs of *T. brasiliensis* at 24, 48, 72, 96 and 120 h post-blood meal on outbred dogs treated (*n* = 4) with fluralaner (Bravecto^®^) at 1, 2, 3, 4, 5, 6, 7, 8, 9, 10, 11 and 12 months post-treatment

We analyzed the mortality of triatomines up to 5 days after each blood meal, which is the length of time required for the elimination of metacyclic trypomastigotes in the feces and/or urine of the insects. All triatomines exposed to fluralaner (Bravecto^®^)-treated dogs in the first and second months after treatment died within 1 day post-exposure (Fig. 3a, b). The time needed to reach 100% mortality of triatomines gradually increased to 2 days at the third month and to 5 days at fourth, fifth, sixth and seventh months after treatment of the dogs with  fluralaner (Bravecto^®^) (Fig. 3c–g). Triatomine mortality observed 5 days post-blood meal was 66% in month 8, 57% in month 9, 35% in month 10, 10% in month 11 and 0% in month 12 (Fig. 3h–l).

### The nutritional status of the dog does not influence fluralaner (Bravecto^®^) insecticidal activity against ***T. brasiliensis.***

**Fig. 4:**
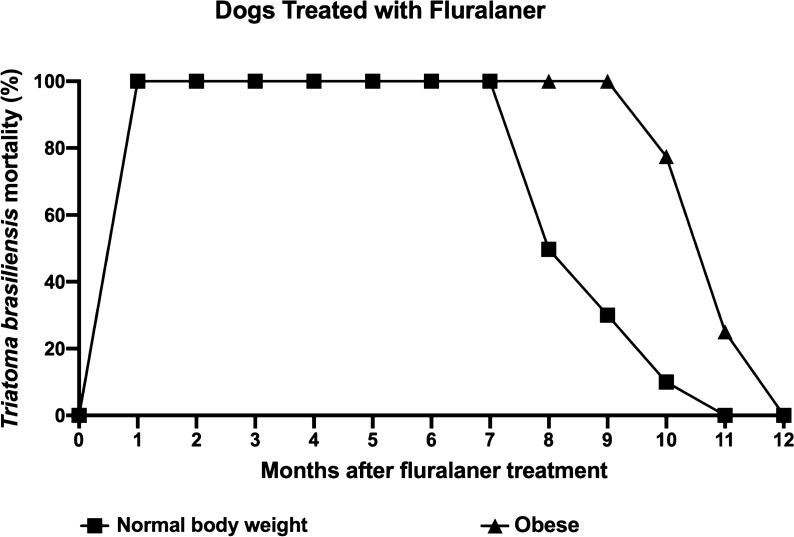
Insecticidal effect of fluralaner (Bravecto^®^) against *T. brasiliensis* is not influenced by the dog’s body mass index. Mortality proportion (%) of third-, fourth- and fifth-instar nymphs of *T. brasiliensis* at 120 h post-blood meal on outbred dogs treated with fluralaner (Bravecto^®^) with normal body mass index (*n* = 2) and with high body mass index (*n* = 2) at months 1–12 post-treatment

The fluralaner (Bravecto^®^)-treated dogs were subclassified as obese or normal body weight to analyze whether the dog’s nutritional status would influence the insecticidal effect of  fluralaner (Bravecto^®^). Mortality among triatomines after blood-feeding on fluralaner (Bravecto^®^)-treated dogs was 100% for both groups (obese and normal-weight dogs) between 1 and 7 months after the fluralaner (Bravecto^®^) treatment. In the following months, triatomine mortality differed between the two groups, remaining at 100% mortality in treated obese animals in months 8 and 9 (vs 49.7 and 30% mortality, respectively, in normal-weight dogs), then falling to 77% (vs 10% in normal-weight dogs) in month 10 and to 25% (vs 0% in normal-weight dogs) in month 11. None of the insects died after a blood meal in both groups in month 12 (Fig. 4). These data show that the dog’s nutritional status does not significantly influence the insecticidal efficacy of fluralaner (Bravecto^®^) against *T. brasiliensis* after blood-feeding on treated-dogs during the follow-up (GLMM: *P* > 0.001) (Table 1).

## Discussion

In this study, we demonstrated that a single oral dose of fluralaner (Bravecto^®^) to dogs induces high *T. brasiliensis* mortality after a blood meal on treated dogs. Insecticidal efficacy was observed for up to 10 months after treatment, irrespectively of the animal’s body weight, suggesting that treating dogs with fluralaner is an a control strategy for Chagas disease in endemic areas.

The mortality of *T. brasiliensis* was 100% within 120 h post-blood meal on treated dogs for up to 7 months. Susceptibility to fluralaner (Bravecto^®^) was determined in *T. infestans* in Bolivia [45] and Argentina [46], where promising results were observed. The study conducted in Bolivia evaluated the insecticide activity of three commercial, oral, single-dose insecticides, namely fluralaner (Bravecto^®^), afoxolaner (NexGard^®^) and spinosad (Comfortis^®^), in outbred dogs exposed to blood-feeding by third- and fourth-instar nymphs of *T. infestans*. The authors of that study observed 100% mortality of *T. infestans* up to 51 days after treatment with fluralaner (Bravecto^®^) and afoxolaner (NexGard^®^) [45]. The study conducted in Argentina assessed the insecticide efficacy of a single oral dose of fluralaner (Bravecto^®^) administered to dogs against pyrethroid-resistant and -susceptible *T. infestans* (third- and fifth-instar nymphs). Cumulative bug mortality in pyrethroid-resistant and -susceptible insects was 100% up to 2 months after dog treatment, decreasing to approximately 80% in months 3 and 4 and to 0–2% at 7 months [46]. These results suggest that *T. brasiliensis* is more susceptible to the insecticidal action of fluralaner (Bravecto^®^) administered in dogs when compared to *T. infestans*.

In the present study, we analyzed the mortality of *T. brasiliensis* up to 120 h after the blood meal. This is the period needed for the metacyclic trypomastigotes to be released by triatomines [49–53]. The interval between triatomine infection and trypomastigote excretion is important with respect to food contamination and also to prevent vectorial infection of another vertebrate host. In this study, bugs intoxicated after the blood meal on fluralaner (Bravecto^®^)-treated dogs were immobile, lying with their backs on the ground, which greatly reduces the chance of vectorial and oral transmission. Release of metacyclic trypomastigote forms of *T. cruzi* after triatomine infection is dependent on temperature, triatomine species, parasite strain, inoculum, mixed or single strain infection, immune factors, the microbiota and nutritional status of the insect [49–51, 54]. *Rhodnius prolixus* infected with two *T. cruzi* isolates (Tc-I and Tc-II) in single and mixed infections started to release metacyclic trypomastigotes at 5 days after infection [49]. Tamayo et al. demonstrated the presence of metacyclic trypomastigotes in rectal ampulla of *R. prolixus* at 6 and 16 days after infection with the TcI and TcII discreet typing units (DTU) of *T. cruzi* strain, respectively [51]. The time required to release metacyclic trypomastigote forms in the feces and urine of *T. brasiliensis* has not yet been determined.

Interestingly, when we analyzed the efficacy of fluralaner (Bravecto^®^) at 96 h post-blood meal in dogs, we observed 100% mortality of *T. brasiliensis* in months 1, 2 and 3 post-treatment and 62.5 and 33% in months 4 and 7 post-treatment, respectively. Data in the literature demonstrate cumulative *T. infestans* mortality of 100, 100, 79–80%, 70–77% and 2% at months 1, 2, 3, 4 and 7 post-treatment, respectively [46]. Interestingly, we observed that the insecticidal effect of fluralaner (Bravecto^®^) was similar in obese and normal body weight animals during the follow-up. Fluralaner (Bravecto^®^) is well distributed in dogs through tissues after being absorbed; however, the highest concentrations have been found in fat, followed by liver, kidney and muscle [34]. The greater concentration of fluralaner in fat tissue may prolong the insecticide action in obese dogs.

The insecticidal activity of fluralaner (Bravecto^®^) on *T. brasiliensis* and *T. infestans* indicates that this approach of treating dogs could contribute to Chagas disease control in Latin America. Based on the results of the present study, we recommend that fluralaner (Bravecto^®^) be administered to dogs from endemic areas every 7 months to interrupt *T. cruzi* transmission. After the seventh month, 100% insect mortality is not achieved, but the effective time period may allow the maintenance of the parasite transmission in endemic areas. *Triatoma brasiliensis* is the triatomine species of greatest importance in the domestic transmission of *T. cruzi* in the Caatinga biome, being the main vector in Brazil [4, 55, 56]. Dogs infected with *T. cruzi* have been identified in several cities of Rio Grande do Norte state, likely contributing to parasite transmission by the oral and vector routes [16]. Several cases of acute Chagas disease were recently demonstrated in Rio Grande do Norte as being due to the ingestion of sugarcane juice contaminated with *T. brasiliensis*. Dogs were identified as reservoirs of the parasite [16, 57].

Another relevant aspect is the occurrence of mixed infection with *T. cruzi* and *Leishmania* spp. in dogs in Latin America because there is an overlap of endemic areas for Chagas disease and cutaneous and visceral leishmaniasis [5, 30, 58–61]. Fluralaner (Bravecto^®^) treatment of dogs also has an insecticidal effect on sand flies [37], suggesting that it this treatment could possibly assist in reducing the canine infection rate by *T. cruzi* and *Leishmania* spp. in endemic areas.

## Conclusions

The present study demonstrates that fluralaner (Bravecto^®^) induces long-term mortality of *T. brasiliensis* after a blood meal in treated dogs and can be used as a potential method to control vectorial transmission of *T. cruzi*, the etiological agent of Chagas disease.

## Data Availability

Data supporting the conclusions of the present study are included within the article. Data used and/or analyzed during this study are available from the corresponding author upon reasonable request.

## Abbreviations

ZCC: Zoonoses Control Center
SD: Standard deviation
